# Editorial: Exploring the psychology of vocational education: from the perspective of literacy promotion, volume II

**DOI:** 10.3389/fpsyg.2025.1640884

**Published:** 2025-07-09

**Authors:** Jian-Hong Ye, Mei-Yen Chen, Yung-Wei Hao

**Affiliations:** ^1^Faculty of Education, Beijing Normal University, Beijing, China; ^2^National Institute of Vocational Education, Beijing Normal University, Beijing, China; ^3^Graduate Institute of Sport, Leisure and Hospitality Management, National Taiwan Normal University, Taipei, Taiwan; ^4^Graduate Institute of Curriculum and Instruction, National Taiwan Normal University, Taipei, Taiwan

**Keywords:** career development, construction of the core literacy, occupational quality, professional competence, vocational education psychology, vocational education and training (VET)

As an important area of support within vocational and technical education and training, vocational education psychology offers valuable theoretical and practical insights into the psychological characteristics, motivational factors and career development of learners within vocational education settings. As well as focusing on cultivating students' vocational skills, it explores how psychological interventions and educational strategies can help students adapt to the vocational environment, enhance their vocational qualities, and achieve a balance between their personal and professional lives. Vocational education psychology emphasizes personal growth and self-actualisation in students' career development processes, arguing that students can only achieve satisfaction and a sense of accomplishment in their careers, and maximize their personal value, when their interests, abilities, and values align with career requirements. As a result, research in the field of vocational education psychology is gaining increasing attention.

In the years leading up to 2020, the focus of vocational education psychology research was on cultivating students' literacy, the effectiveness of innovative teaching methods and technology-assisted skills learning, all examined from a psychological perspective. Between 2020 and 2023, the pandemic imposed physical constraints, prompting experts in the field to focus on its effects on students' online learning, mental health, and wellbeing. In the post-epidemic era, research in this field has shifted toward students' vocational literacy, AI literacy, digital literacy and career development. The issue of mental health and wellbeing of teachers and students in vocational colleges and universities has also received a great deal of attention in the context of the Sustainable Development Goals (SDGs).

The rapid development of new metaverse technologies such as artificial intelligence, virtual reality, cloud computing, digital life, big data and blockchain is driving the Fourth Industrial Revolution. This will result in many repetitive and empirical work tasks being replaced by robots and robotic arms, and summarizing and generalizing work tasks being replaced by artificial intelligence. This digital transformation will also change the demand for talent in the labor market. Consequently, more attention will be paid to research on career adaptability. Future research will focus on enhancing students' career adaptability through education and training to help them transition smoothly from school to work in a rapidly changing labor market. The practical effectiveness of career adaptation interventions will also be a research focus.

As contemporary workplaces demand increasingly high-quality standards from their employees, it is important to recognize that a highly educated workforce will be better equipped to meet these demands. Highly educated professionals can adapt more easily to changes in the work environment and meet enterprises' comprehensive quality requirements. They are also more likely to have promotion opportunities and career development. Therefore, as the vocational education system accelerates in development, the issue of cultivating high-quality vocational education personnel has become important. This has made vocational education face unprecedented challenges, introducing new requirements for the objectives, qualities, content, methods and assessment standards of personnel training.

In addition to making vocational education students more competitive in the job market, it is also necessary to pay more attention to their career development, helping them to understand their interests, abilities and vocational inclinations, and to plan their careers effectively. As digitalisation and globalization develop, the integration of technology will become an important area of research in vocational education psychology. These emerging technologies will be deeply integrated into all aspects of vocational education, and vocational education psychology research will focus on meeting students' needs through innovative educational models. For instance, artificial intelligence can assist with teaching and learning and enhance diagnostic efficiency through emotion recognition technology. Meanwhile, virtual reality technology can simulate real workplace scenarios to help students adapt more easily to future professional environments. Intelligent counseling systems will enable personalized learning paths to be planned. Furthermore, the study will focus on changes to the content of vocational education programmes to ensure they better meet the needs of the future job market.

The OECD's PISA 'Vocational Education and Training (VET): A Framework for Evaluation and Analysis' is an important cross-national research project. It enriches the theoretical system of vocational education psychology and provides a scientific basis for formulating vocational education policies in various countries. This is helping educators to better understand students' needs and to design curricula and teaching methods that promote vocational literacy development. This is improving the accuracy of assessing the quality of vocational education in various countries.

There are a wide range of recent research themes in vocational education psychology, including those conducted in the vocational education setting: the TVET programme and health-related quality of life (Yasin et al.), occupational psychological quality and mental health (Luo et al.), occupational barriers and career readiness behaviors (Shin and Ra), influences on task motivation (Ma and Chen), anxiety, depression, and sleep during the normalization of COVID-19 outbreak management (Gao et al.), school support and innovativeness (Fongkanta and Buakanok), and teacher resilience (Duan et al.), to name a few of the rich explorations.

Of course, Research Topics in vocational education psychology are not limited to the aforementioned themes. Future research in this field will need to explore additional theoretical models or frameworks based on vocational education contexts in order to better explain phenomena within it. Therefore, research in vocational education incorporating perspectives from various disciplines, such as educational and sociological theory, management, psychology and philosophy, will continue to innovate and evolve.

As the field of vocational education develops and expands, the Research Topics in vocational education psychology are diversifying, with an increasing number of research hotspots, far exceeding the traditional recognized themes. Future research in this field must closely align with the context of vocational education, delving deeper to construct more targeted, forward-looking and scientifically sound theoretical models or frameworks. This will enable more precise and comprehensive explanations of the various complex phenomena and issues emerging in vocational education.

Consequently, research on vocational education psychology from various disciplinary perspectives, such as education, sociology, law, management, psychology and philosophy, will continue to innovate and contribute to the ongoing development of vocational education psychology. This will drive the field toward greater maturity and perfection, thereby better serving the high-quality development of vocational education.

